# Investigation on the Continuous Wave Mode and the ms Pulse Mode Fiber Laser Drilling Mechanisms of the Carbon Fiber Reinforced Composite

**DOI:** 10.3390/polym12030706

**Published:** 2020-03-23

**Authors:** Xiao Li, Wentao Hou, Bing Han, Lingfei Xu, Zewen Li, Pengyu Nan, Xiaowu Ni

**Affiliations:** 1 School of Science, Nanjing University of Science & Technology, Nanjing 210094, China; gslslhl@163.com (X.L.); nust_nan@163.com (P.N.); nxw@njust.edu.cn (X.N.); 2 MIIT Key Laboratory of Advanced Solid Laser, 2011 Co-innovation Center, Nanjing University of Science & Technology, Nanjing 210094, China; lizewen@njust.edu.cn; 3 Shanghai Academy of Spaceflight Technology, Shanghai 201109, China; houwentao@sast.cn; 4 Joint International Research Laboratory of Laser-based Manufacturing and Material Processing, Nanjing University of Science & Technology, Nanjing 210094, China; 5 Shanghai Institute of Electrical and Mechanical Engineering, Shanghai 200030, China; jsj900@163.com

**Keywords:** laser drilling, ms pulse, peak power density, surface HAZ, mechanical erosion

## Abstract

The near infrared (NIR) laser drilling of a carbon fiber reinforced polymer (CFRP) composite in the continuous wave (CW) mode and the ms pulse mode was investigated by an experiment and a numerical simulation. The relationships between the laser penetrating time, entrance hole diameter, surface heat affected zone (HAZ) width, and material ablation rate and the laser irradiation time and laser peak power densities were obtained from the experiment. For the same average power density of the laser output, 3.5 kW/cm^2^, it was found that the ms pulse laser mode, which had a higher peak power density, had a higher drilling efficiency. When drilling the same holes, the pulse laser mode, which had the highest peak power density of 49.8 kW/cm^2^, had the lowest drilling time of 0.23 s and had the smallest surface HAZ width of 0.54 mm. In addition, it was found that the laser penetrating time decreased sharply when the peak power density was higher than 23.4 kW/cm^2^. After analyzing the internal gas pressure by the numerical simulation, it was considered that a large internal gas pressure appeared, which resulted from polymer pyrolysis, causing a large amount of the mechanical erosion of the composite material to improve the drilling efficiency. Therefore, the ms pulse laser showed its potential and advantage in laser drilling the CFRP composite.

## 1. Introduction

Fiber-reinforced polymer composite materials are used for engineering applications where toughness, durability, corrosion resistance, abrasion resistance, and thermal stability are needed. In particular, carbon fiber reinforced polymer (CFRP) composites with superior structural capabilities are lightweight and have been widely used, particularly in the aerospace and automotive industries. Unfortunately, the conventional machining of CFRPs results in high tool wear and short tool life, which, in turn, produces high costs, as well as machining quality issues [1,2]. Lasers, as non-contact, fast, precise, and flexible tools, have been successfully used for the processing of both metallic and non-metallic materials. However, the thermal loading from the near infrared (NIR) laser processing of CFRPs may be a major concern with potential industrial users due to the anisotropic and heterogeneous properties of CFRPs.

CFRPs consist of carbon fibers and a resin matrix. The sublimation temperature of the carbon fibers is approximately 3600 K, whereas the sublimation temperature of the resin matrix is approximately 800 K. Due to the great difference in the thermal properties of the carbon fibers and the resin matrix, the energy required to sublimate carbon fiber is more than 50 times the energy required to sublimate polymer resin for the same volume case. In addition, the thermal conductivity of carbon fiber is about 250 times that of resin in the direction of the parallel fiber axis. Therefore, carbon fibers cannot be ablated with the laser energy designed to ablate the resin. In addition, the use of more laser energy to ablate the carbon fibers could cause the recession and decomposition of the excess matrix, resulting in the presence of an extended heat affected zone (HAZ). In the HAZ, fiber swelling [3,4], thermal degradation of the polymer resin [5], matrix delamination [6], and the strength decline of the material [7] might appear, which could damage the structural properties of the CFRPs. At present, the reduction of the HAZ during the laser processing of CFRPs has been the focus of researchers.

To reduce the HAZ, ultraviolet (UV) lasers [8,9] and NIR short pulse lasers [6,10,11] were used to minimize the thermal effect. However, a major drawback of these laser systems is their limited laser power, which results in a low processing efficiency. In order to find better laser processing parameters, some research groups have already done some work on the subject of laser cutting by continuous wave (CW) lasers and ms pulse lasers [12,13,14]. The results showed that the NIR lasers of the CW mode and the ms pulse mode had great potential advantages in reducing laser processing time. In addition, it was proven that a ms pulse laser was superior to a CW laser for the same laser power density for laser cutting [12,13]. This was because there was a higher laser intensity from the pulse mode, which meant that there was a shorter cutting time, providing a smaller thermal load to help obtain a narrower HAZ and a better cutting quality. Therefore, the ms laser was expected to reduce the processing time and ensure the processing quality.

In contrast, the research on laser drilling CFRPs have recently mainly focused on UV short pulse (ns) lasers [8,15,16] and NIR ultrashort pulse (fs-ps) lasers [11,17]. At present, few studies about ms pulse laser drilling with a high pulse repetition rate have been reported. In this work, we investigated the performance of the 1080 nm fiber laser drilling of a carbon fiber/epoxy composite in CW mode and in ms pulse modes with an experiment and with a numerical simulation. The drilling times, entrance hole diameters, surface HAZ widths, and material ablation rates for different laser modes were compared and discussed.

## 2. Experimental Methods

### 2.1. Material Specification

The sample size of the CFRP panels was 60 mm × 60 mm × 1 mm. The panels were made from five plies of carbon fiber cloth (T300) encapsulated in an epoxy matrix. A plain-woven structure was used in the carbon fiber cloth (50% fiber in the warp direction) of the two plies on the composite surface, and a unidirectional structure was used in the carbon fiber cloth of the three plies in the middle. The ply orientation was 0°/90°. The thickness of each ply was about 0.2 mm, and the diameter of the carbon fiber was about 6 μm. The average fiber volume of the composite samples remained about 70%. The thermal properties of the carbon fibers and the matrix are shown in Table 1 [18].

### 2.2. Laser Setup

**Figure 1:**
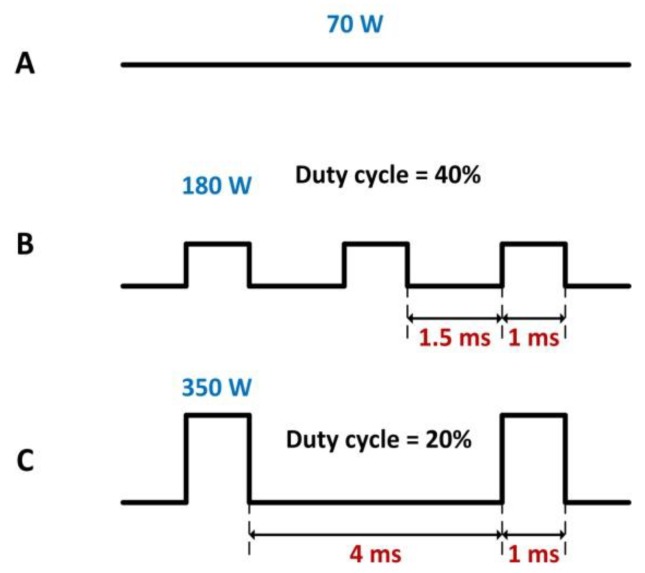
Temporal profiles of the three laser working modes: (**A**) The continuous wave (CW) mode with an output power of 70 W. (**B**) The pulse mode with a repetition rate of 400 Hz, pulse width of 1 ms, and pulse peak power of 180 W. (**C**) The pulse mode with a repetition rate of 200 Hz, pulse width of 1 ms, and pulse peak power of 350 W. For different laser modes, the laser source had the same average output power of 70 W.

A fiber optic-delivered 1000 W laser, which emitted at 1080 nm wavelength, was used in the experiment. In each test, the thickness of the CFRP specimen was 1 mm, and the target specimen was fixed on a two-axis translation stage that could move in the directions of vertical laser incidence. The laser beam was focused with a lens that had a 250 mm focal length on the surface of the specimen and a Gaussian laser intensity distribution was provided. The diameter of the spot on the specimen surface was fixed at 1.6 mm, and the entire target specimen was in front of the focus of the laser beam. Using an electric control switch, the laser source could operate for different modes of CW and ms pulses. The temporal profiles of several laser modes are shown in Figure 1. In the CW mode, the laser power used was fixed at 70 W. In the ms pulse modes, the pulse width was fixed at 1 ms. The range of other laser parameters utilized is summarized in Table 2. In each laser mode, the average output power of the laser source was maintained at 70 W (69.6–72 W).

### 2.3. Time Measurement of the Laser Penetrating Target

**Figure 2:**
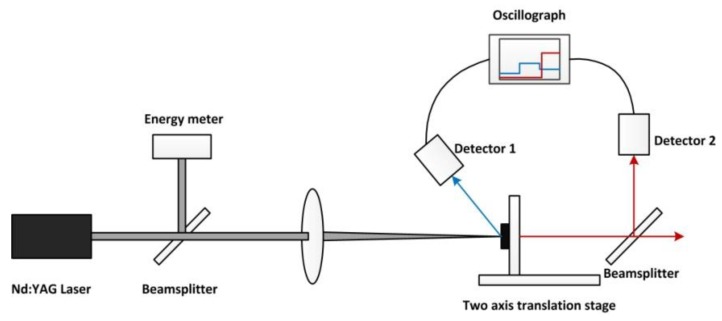
Schematic of the experimental system.

A schematic of the experimental system for measuring the penetrating time of the laser drilling through the target specimen is shown in Figure 2. When the laser beam reached the surface of the specimen, part of the scattered light signal was received by Detector 1. When the laser beam drilled through the target specimen, part of the reflected light signal was received by Detector 2. According to the signal curves of Detector 1 and Detector 2 shown in the oscillograph, the time that was needed to penetrate the target specimen during the laser drilling could be gained.

### 2.4. Measurement of the Entrance Hole Diameter and the Surface HAZ Width

**Figure 3:**
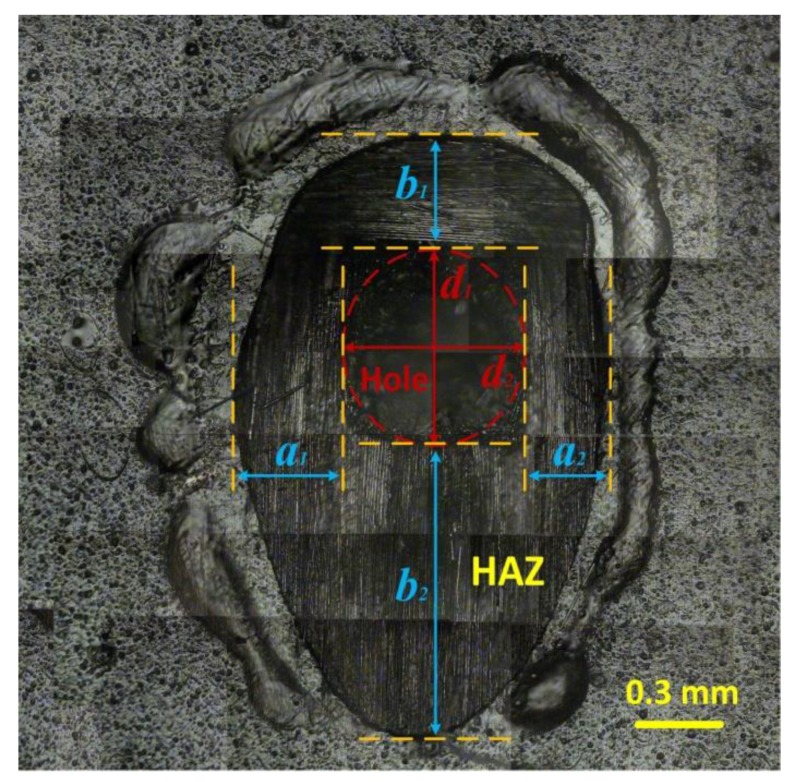
Morphology of the specimen surface after laser drilling.

A metallurgical microscope was used to observe the surface morphologies of the drilled specimens. In Figure 3, the region of ablated fibers (marked with red dotted line) was the entrance hole, and the region of fibers that protruded from the epoxy matrix was the surface HAZ [6,9,19]. For the same laser parameters, the sizes and shapes of the entrance holes were almost invariable. The shape of the entrance hole approximated the shape of a standard circle. However, due to the plain-woven structure of the carbon fiber cloth, the composite surface had an anisotropic heat conduction. Therefore, the sizes and shapes of the surface HAZs might significantly change with the laser irradiation positions on the specimen surface. In order to accurately describe the change rule of the surface morphologies, there were no less than 20 drilled holes tested for every laser condition. Additionally, unified calculation methods were provided for the entrance hole diameter ($D_{entra}$) and the surface HAZ width ($W_{HAZ}$). The $D_{entra}$ and the $W_{HAZ}$ could be calculated as follows:

(1) $$D_{entra}=\frac{d_{1}+d_{2}}{2},$$

(2) $$W_{HAZ}=\frac{a_{1}+a_{2}+b_{1}+b_{2}}{4},$$

where $d_{1},\;\;d_{2},\;\;a_{1},\;a_{2},\;b_{1},\;b_{2}$ could be measured by the metallurgical microscope with a resolution of 0.1 μm, as shown in Figure 3.

### 2.5. Mass Measurement of the Ablated Material

The mass of material ablated by laser drilling could be measured by an electronic balance. First, a clean cloth was used to wipe the specimen surface with an alcohol-water blend. Second, the specimen was weighed with an electronic balance whose accuracy was up to 0.1 mg. Third, the laser drilling test was carried out 4–7 times at different positions on the specimen surface. After laser drilling, the specimen surface was wiped with the alcohol-water blend again to remove the splashes attached to the edge of the holes. Then, the specimen was weighed again to calculate the mass of the ablated material.

## 3. Experimental Results and Discussion

### 3.1. Laser Penetrating Time

**Figure 4:**
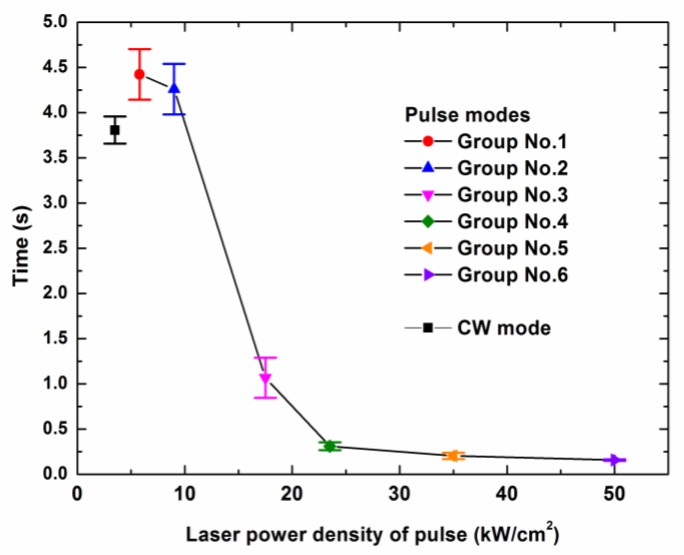
Mean penetration time dependence on the peak power densities of different laser modes. The laser parameters for the different laser modes are shown in Table 2.

The mean penetration times of the laser drilling through the target specimen with different laser modes are shown in Figure 4. For each laser mode, no less than seven samples were tested. It was revealed that the mean penetration times in the ms pulse modes were less those that in the CW mode, when the peak power densities of the laser pulses were much larger than the average output power density of 3.5 kW/cm^2^. In the ms pulse modes, the penetration time increased with the decrease of the peak power density and increase of the pulse frequency. For Group No. 1 and Group No. 2, for which the peak power densities were similar to the mean output power density, it could be seen that the penetration time was greater than that of the CW mode.

By neglecting the thermal dissipation from the thermal conduction, surface radiation, and surface convection cooling of the composite, the laser penetration time could be calculated as follows [20]:

(3) $$t_{Laser}=\frac{\rho (L_{v}+C_{p}\left(T_{v}-T_{0}\right))h}{2I_{0}\tau },$$

where ρ*,* $L_{v}$*,* $C_{p}$*,* h*,* $I_{0}$*,* $T_{v}$, $T_{0}$, and τ are material density, latent heat of sublimation, heat capacity, material thickness, laser peak power density, gasification temperature of material, initial temperature of material, and duty cycle, respectively. For the CW mode, τ=1. The detailed description of Equation (3) is given in the Appendix A.1 of the Appendix A.

For the ms pulse modes, the number of required laser pulses could be calculated using the following expression:

(4) $$N=\frac{\tau t_{Laser}}{t_{pulse}}$$

where $t_{pulse}$ = 1 ms is the pulse duration and $\frac{\tau }{t_{pulse}}$ represents the pulse repetition frequency.

From Equation (3), it could be found that a higher laser power density required a shorter time to drill through the material for the CW mode. For the ms pulse modes used in the experiment, the $t_{Laser}$ calculated by Equation (3) was the same. However, Equation (4) revealed that a smaller number of the laser pulses was required in order to drill through the material when the laser pulse had a higher peak power density. In fact, an amount of heat was taken away by the thermal conduction of the carbon fibers in the interaction time between the laser beam and the target material. Although the laser energy irradiation on the target surface in unit time was the same for different laser modes, the pulse laser mode, which had a lower peak power density and a higher pulse frequency, required a longer interaction time. That led to more thermal energy being taken away through the thermal conduction. This could explain why the laser penetration time decreased with the increase of the peak power density, as shown in Figure 4. Of course, the CW mode had the longest interaction time. However, considering the competition between the thermal deposition and the thermal dissipation, it was reasonable that the laser penetration time of the CW mode was less than that of the pulse modes of Group No. 1 and Group No. 2. 

However, a new issue could be found in the fact that there was an abrupt change of the slope of the curve in Figure 4 that appeared before and after the point of 23.4 kW/cm^2^. It was difficult to imagine that this strong change was caused by the same thermal ablation mechanism. Therefore, the $t_{\mathrm{Laser}}$ was calculated by using Equation (3), in which $\rho^{fiber}$ = 1850 kg/m^3^, $L_{v}$ = 43,000 kJ/kg, $c_{p}{}^{fiber}$ = 710 J/(kg·K), $T_{v}{}^{fiber}$ = 3600 K, $T_{0}$ = 300 K, h = 0.7 mm. Because the thermal conduction from the carbon fiber was neglected and only the sublimation mechanism of the carbon fiber ablation was considered, the calculated $t_{Laser}$ had to be smaller than the experimental values of the mean penetration time if the sublimation was the main thermal ablation mechanism during the laser drilling.

Based on the calculation, $t_{Laser}$ was about 840 ms, which was larger than the mean penetration times of Group No. 4–6 in the experiments. This showed that there was at least one other ablation mechanism in addition to sublimation during the laser drilling. This unknown ablation mechanism was especially significant for the ms pulse laser drilling with a high peak power density. This issue is discussed further in Section 4.1.

### 3.2. Entrance Hole Diameter and Surface HAZ Width

**Figure 5:**
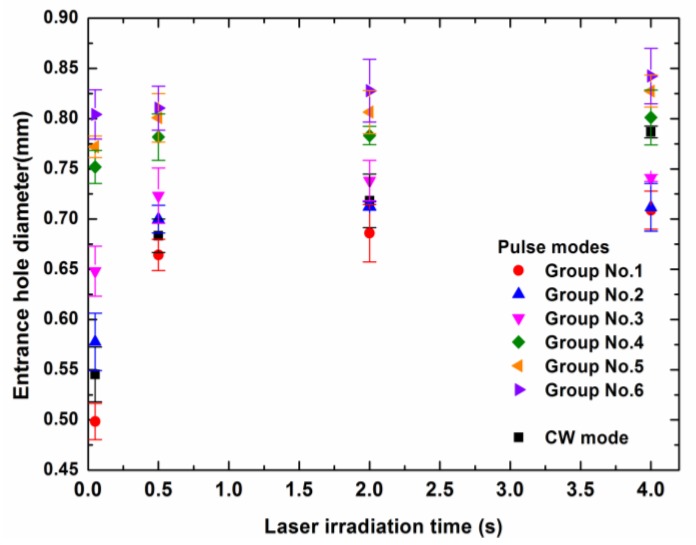
Entrance hole diameter dependence on the laser irradiation time for different laser modes. The laser parameters for the different laser modes are shown in Table 2.

Figure 5 shows the relationship between the entrance hole diameter and the laser irradiation time for different laser modes in the experiment. The laser irradiation times included 0.05, 0.5, 2, and 4 s in the experiment. Additionally, there were no less than 20 drilled holes tested for every laser condition. 

From Figure 5, it was obvious that in the pulse modes, the entrance hole diameter increased with the increase of the peak power density of pulse laser with the same laser irradiation time. This showed that the pulse mode, which had a higher peak power density and a lower pulse frequency, could lead to more thermal energy being deposited in the laser spot rather than dissipated by thermal conduction. Additionally, it could be seen that a lower peak power density led to a larger increase of the entrance hole diameter from 0.05 to 0.5 s. After 0.5 s, the entrance hole diameter in all of the pulse modes tended to be gentle. The experimental result showed that a ms pulse mode, which had a lower peak power density, led to a smaller entrance hole diameter even if the composite was drilled for a long time.

For the CW mode, the interaction time of the laser beam and the composite surface was the longest in all of the laser modes. A large amount of heat was transferred from the center of the spot to the outside by the thermal conduction of carbon fibers, which helped to increase the entrance hole diameter when the irradiation time was long enough.

**Figure 6:**
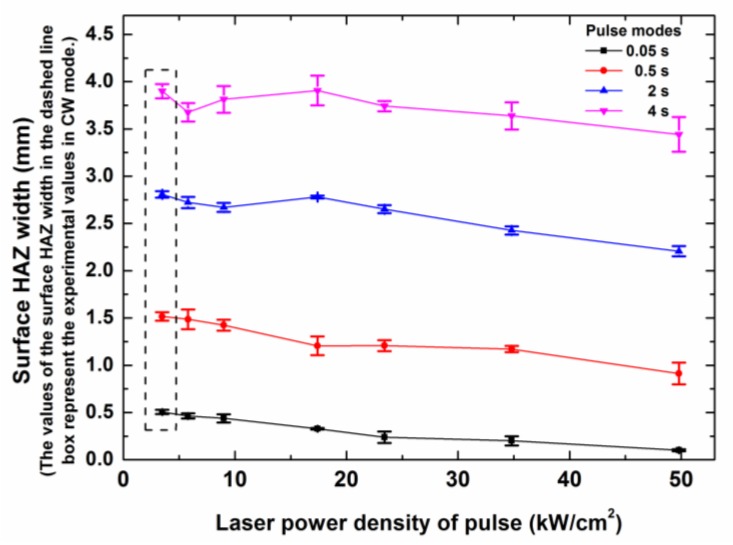
Surface heat affected zone (HAZ) width dependence on the laser peak power densities of different laser modes with different laser irradiation times. The laser parameters for the different laser modes are shown in Table 2.

The relationship of the surface HAZ width, the different laser modes, and the laser irradiation time is shown in Figure 6. With the increase of the irradiation time, the surface HAZ width increased obviously. This was because there was a large amount of thermal energy, which was not enough to ablate the carbon fibers but which could ablate the epoxy matrix. This thermal energy was transferred to the surroundings by the thermal conduction of carbon fibers. With the increase of the laser irradiation time, more thermal energy diffused from the center to the outside, which caused an increasing surface HAZ width.

For the same irradiation time, the surface HAZ width mainly decreased with the increase of the laser peak power density, which was opposite to the dependence of the entrance hole diameter on the laser peak power density. The fiber ablation decreased the interaction time of the laser beam and the surface material, which weakened the thermal diffusion of the composite surface. Therefore, a laser mode that had a higher laser power density led to a smaller surface HAZ width for the same irradiation time. Some anomalous points of the nonmonotonic curves in Figure 6 were attributed to the obvious influence of the surface convection cooling in the pulse modes, which had low peak power densities.

### 3.3. Material Ablation Rate

**Figure 7:**
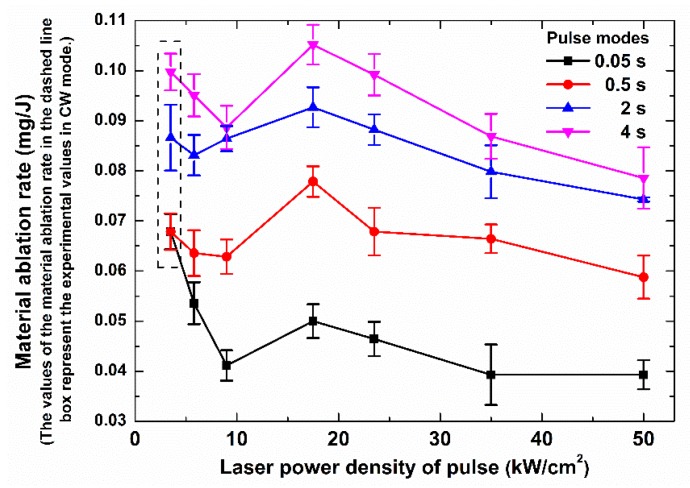
Relationship for the material ablation rate, the laser irradiation time, and different laser modes. The laser parameters for the different laser modes are shown in Table 2.

Figure 7 shows the relationship for the material ablation rate, the laser irradiation time, and different laser modes. The irradiation times included 0.05, 0.5, 2, and 4 s. There were no less than five samples, including four to nine drilled holes, tested in every laser condition. The material ablation rate was defined as the mass loss divided by the laser energy emitted by the laser source in every test. When the laser irradiation time was 0.05 s, the ms pulse mode that had a higher peak power density led to a lower material ablation rate. This was because the higher peak power density meant that there was more absorbed laser energy being used for carbon fiber ablation rather than epoxy matrix ablation. For the carbon fibers and epoxy matrix of the same mass, much more energy was required to ablate the carbon fibers than the epoxy matrix. Hence, a higher percent of the laser energy used for the carbon fiber ablation led to a lower material ablation rate. This could explain why the material ablation rate of the CW mode was far larger than the material ablation rate of the pulse modes at the laser irradiation time of 0.05 s.

With the increase of the laser irradiation time, the material ablation rate in each laser mode increased, as shown in Figure 7. For the pulse mode of Group No. 6 (49.8 kW/cm^2^, 72 Hz), in which the target specimen was drilled through earlier than it was for the other laser modes, there was more laser energy loss with the laser beam passing through the target directly from the exit hole. Therefore, the material ablation rate of Group No. 6 was the lowest in the range of 0.5 to 4 s. For the pulse modes of Group No. 1 (5.8 kW/cm^2^, 600 Hz) and Group No. 2 (9 kW/cm^2^, 400 Hz), in which the target specimen was drilled through more slowly than it was for the other laser modes, the thermal energy transfer in thickness direction of the composite was slower than those of the other laser modes were. Hence, the inner matrix could not be heated for ablation, and no more of the outer polymer matrix could be ablated. Therefore, their material ablation rates were in the middle in the range of 0.5 s to 4 s. The material ablation rate in the pulse mode of Group No. 3 (17.4 kW/cm^2^, 200 Hz) was the maximum in the range of 0.5 s to 4 s. The maximum material ablation rate corresponded to the pulse mode in which the slope of the laser penetrating time curve started to change, as shown in Figure 4. Because the drilling mechanism started to change, the drilling speed in the pulse mode was in the middle of the range of all of the drilling speeds. This showed that a suitable drilling speed was helpful for increasing the material ablation rate and led to a large HAZ inside the composite. Therefore, for the quality of laser drilling, the pulse mode, which had a higher peak power density, was useful for decreasing the HAZ on the surface and in the composite with a lower material ablation rate.

## 4. Numerical Simulation and Discussion

### 4.1. Simulation of the Internal Gas Pressure

From the experimental result of the laser penetration time described in Section 3.1, it was revealed that there was at least one other ablation mechanism aside from the sublimation during the laser drilling. Considering the amount of pyrolysis gases produced during the polymer matrix ablation, it could be speculated that a huge internal gas pressure appeared in the composite, which caused the mechanical erosion of the carbon fibers or the matrix material. The fragmented material could be ejected from the hole to the composite surface by pyrolysis gases; then, it could either be removed from the surface or ablated by the oxidation reaction. The mechanical erosion of the CFRPs by the pyrolysis gases was experimentally confirmed in the interaction process of the ns pulse laser and the CFRPs [21,22,23].

To prove the speculation, a two-dimensional axisymmetric finite element model was established in order to predict the temperature field and the internal gas pressure in the composite during the laser drilling. This numerical model, which was mainly based on the model by Chippendale et al. [24], considered the processes of the thermal transport, the polymer degradation, the pyrolysis gases transport, and the carbon fiber sublimation in the composite. The model formulations [24,25,26,27,28,29,30,31,32,33,34,35,36] and the calculation parameters [18,24,29] are given in the Appendix A.2 of the Appendix A. The numerical simulation results of the laser drilling the composite in the CW mode and in the pulse modes of Group No. 2 (9 kW/cm^2^, 400 Hz), Group No. 3 (17.4 kW/cm^2^, 200 Hz), and Group No. 5 (34.8 kW/cm^2^, 100 Hz) were compared.

**Figure 8:**
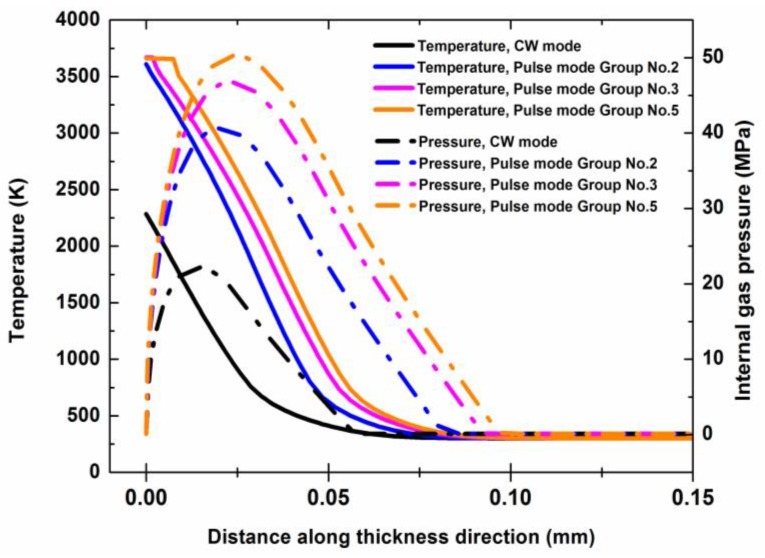
Temperature profile and internal gas pressure profile in the thickness direction for different laser modes with a laser irradiation time of 1 ms. The laser parameters for the different laser modes are shown in Table 2.

Figure 8 shows the temperature field and the internal gas pressure distribution in the composite along the thickness direction with a laser irradiation time of 1 ms (1 pulse duration). From the temperature profiles of Figure 8, it could be found that a higher laser peak power density led to a faster temperature rise and a faster heat transfer in the composite. From the internal gas pressure profiles of Figure 8, it could be found that a higher laser peak power density could cause a larger internal gas pressure in the period of laser heating. Because the shear strength of the carbon fibers used in the composite was about 40 MPa, the carbon fibers in the composite under the pulse modes of Group No. 3 (17.4 kW/cm^2^, 200 Hz) and Group No. 5 (34.8 kW/cm^2^, 100 Hz) were subjected to mechanical erosion at 1 ms. 

**Figure 9:**
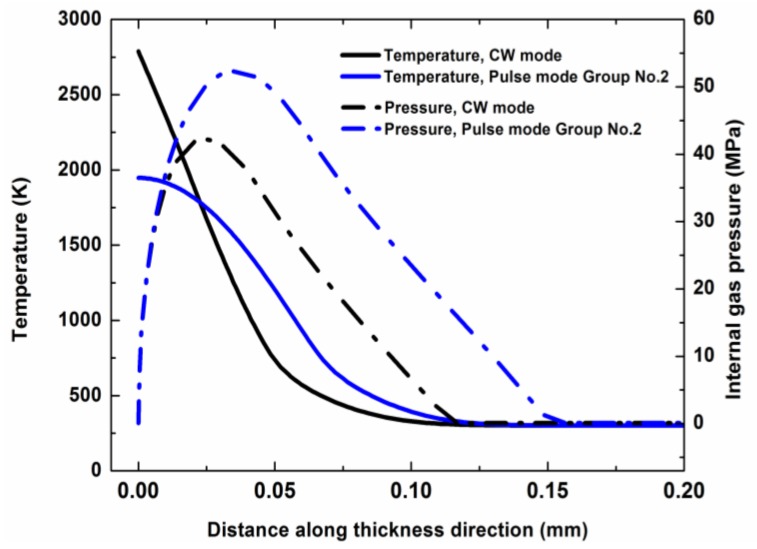
Temperature profile and internal gas pressure profile in thickness direction for different laser modes with a laser irradiation time of 2 ms. The laser parameters for the different laser modes are shown in Table 2.

Figure 9 shows the temperature field and the internal gas pressure distribution in the composite along the thickness direction with a laser irradiation time of 2 ms. For the pulse mode, the laser heating ended at 1 ms and the temperature in the composite started to decrease from 1 ms to 2 ms, as shown in Figure 9. However, the peak of the internal gas pressure profile could continue to rise for a short time before the mechanical erosion appeared. Therefore, for the pulse mode of Group No. 2 (9 kW/cm^2^, 400 Hz), the peak of the internal gas pressure profile could also surpass 40 MPa at 2 ms, as shown in Figure 9. For the CW mode, the laser heating was continuous. Hence, the temperature and the internal gas pressure in the composite could increase slowly with the increase of the laser irradiation time.

Considering the thermal degeneration of the material mechanical strength caused by the rise of the temperature [37], the strength threshold of the mechanical erosion of the composite material decreased with the increase of the temperature. Therefore, a higher laser peak power density could lead to a larger internal gas pressure in the composite at a higher temperature and the internal gas pressure caused more materials, which included the carbon fibers and the matrix, to experience mechanical erosion. It could be concluded that the laser mode, which had a higher laser peak power density, could improve the efficiency of the laser drilling. This could explain the dependence of the laser penetration time on the laser power density for the different laser modes described in Section 3.1 very nicely.

### 4.2. Simulation of the Entrance Hole Diameter and the Surface HAZ Width

The entrance hole of the laser drilling was mainly determined by the thermal properties of the carbon fiber. However, the surface HAZ was mainly controlled by the thermal conduction of the carbon fibers and the thermal properties of the polymer matrix. To accurately predict the entrance hole diameter and the surface HAZ width, a new numerical model, which only considered the process of the thermal transport, was proposed. In this model, the resin matrix layer with a 0.1 mm thickness and the carbon fibers layer with a 0.06 mm thickness were separated. The model formulations [8,32,33,38,39,40] and the calculation parameters [18,24,29] are given in the Appendix A.3 of the Appendix A.

**Figure 10:**
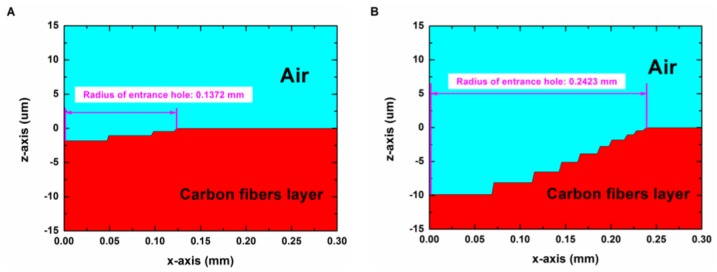
Morphology of the entrance hole in the pulse mode of Group No. 2 (9 kW/cm^2^, 400 Hz) with laser irradiation times of (**A**) 30.7 ms and (**B**) 48.5 ms.

**Figure 11:**
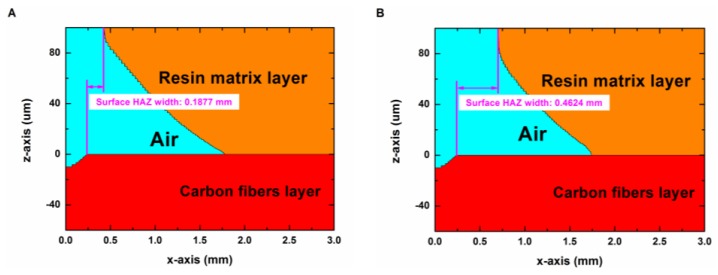
Morphology of the surface heat affected zone (HAZ) in the pulse mode of Group No. 2 (9 kW/cm^2^, 400 Hz) with laser irradiation times of (**A**) 50 ms and (**B**) 77 ms (including cooling for 22 ms).

Because a large amount of computation time and computation space were required in order to simulate the laser drilling process with a long irradiation time, the laser irradiation time in the model varied in the range of 0 to 0.05 s. A part of the simulation results are shown in Figure 10 and Figure 11.

Figure 10 shows the morphology of the entrance hole on the composite surface in the pulse laser mode of Group No. 2 (9 kW/cm^2^, 400 Hz) with laser irradiation times of (**A**) 30.7 ms and (**B**) 48.5 ms. In Figure 10, it can be found that the entrance hole on the surface expanded with the increase of the laser irradiation time, and it reached a maximum at 48.5 ms.

Figure 11 shows the morphology of the surface HAZ of the composite in the pulse mode of Group No. 2 (9 kW/cm^2^, 400 Hz) with laser irradiation times of (**A**) 50 ms and (**B**) 77 ms. From 50 to 77 ms, the composite surface did not receive laser energy and the material was being cooled. From Figure 11, it can be found that the surface HAZ continued to expand in the period without laser irradiation. This was because the matrix layer absorbed the heat, which was from the adjacent high temperature carbon fibers and the surrounding high temperature sublimation gas.

**Figure 12:**
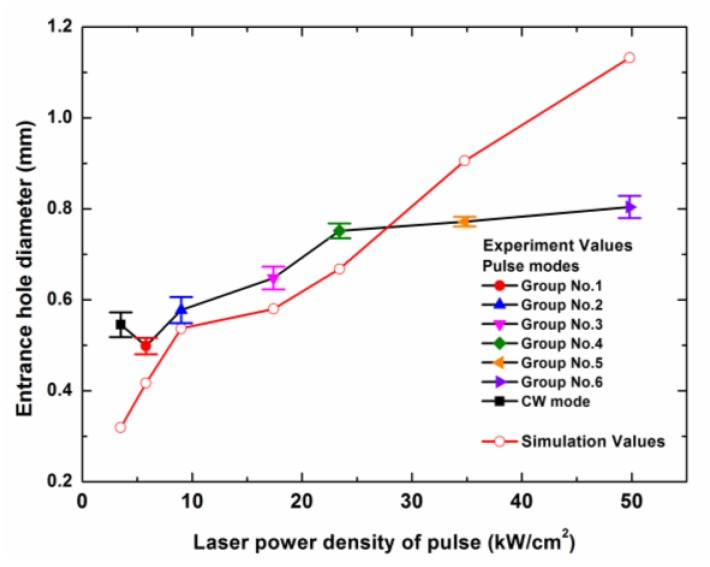
Dependence of the entrance diameters of the experimental values and the simulation values on the different peak power densities of different laser modes with a laser irradiation time of 0.05 s. The laser parameters for the different laser modes are shown in Table 2.

**Figure 13:**
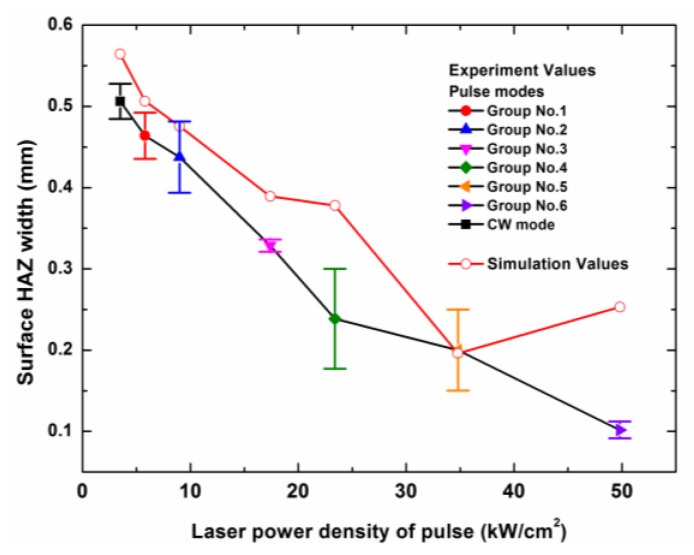
Dependence of the surface heat affected zone (HAZ) widths of the experimental values and the simulation values on the different peak power densities of different laser modes with a laser irradiation time of 0.05 s. The laser parameters for the different laser modes are shown in Table 2.

The experimental results and the simulation results of the entrance hole diameter and the surface HAZ width with a laser irradiation time of 0.05 s were compared, as shown in Figure 12 and Figure 13. From Figure 12, it can be found that from 5.8 kW/cm^2^ to 23.4 kW/cm^2^, the simulation values were similar to the experimental values. When the laser power density was above 23.4 kW/cm^2^, the curve of experimental values tended to be gentle, but the curve of the simulation values continued to rise. This was because the laser beam entered deeper into the composite in the experiment due to the large amount of mechanical erosion of the CFRP material, which was caused by the internal gas pressure in a high laser peak power density. However, in the simulation, the sublimation of the carbon fibers needed a lot of heat so that the laser energy would be deposited on the composite surface during the long time span. Additionally, the thermal energy on the laser center could be transferred to the surroundings by the thermal conduction of carbon fibers, which led to the increase of the entrance hole diameter. In addition, the difference of the entrance hole diameters between the experimental value and the simulation value at 3.5 kW/cm^2^ was attributed to the difference between the numerical model and the reality, such as the simplification of the physical process, the geometry size of the model, the selected parameters, and so on.

Figure 13 shows that the simulation values were similar to the experimental values from 3.5 kW/cm^2^ to 34.8 kW/cm^2^. However, there was a big difference between the simulation value and the experimental value at 49.8 kW/cm^2^. This was because that the interaction time of the laser beam and the composite surface was a short time in the experiment because the mechanical erosion of the CFRP material increased the laser drilling efficiency. Conversely, in the simulation, the interaction time was a longer time because only the sublimation mechanism of the carbon fiber ablation was considered. This led to the larger surface HAZ width of the simulation result at 49.8 kW/cm^2^.

From the simulation result, it could be found that the influence of the internal gas pressure, which could cause mechanical erosion in the composite, on the entrance hole and the surface HAZ could not be ignored when the laser power density was above 34.8 kW/cm^2^. Additionally, the influence of the mechanical erosion on the laser drilling process was difficult to consider in the numerical simulation. When the laser power density was lower than 34.8 kW/cm^2^, the model that only considered the thermal mechanism was able to predict the entrance hole and the surface HAZ of the laser drilling.

## 5. Discussion of the CW Mode and the ms Pulse Modes Laser Drilling Efficiencies

From above experimental results and simulation results, it could be found that it was potentially helpful to improve the processing quality of a drilled hole, decrease the processing time, and reduce the laser energy consumption using a pulse laser mode, which had a high peak power density. To compare the laser drilling efficiencies of the CW mode and the ms pulse modes, the same hole was drilled with the different laser modes, for which the used laser parameters were summarized in Table 2 and Table 3.

**Figure 14:**
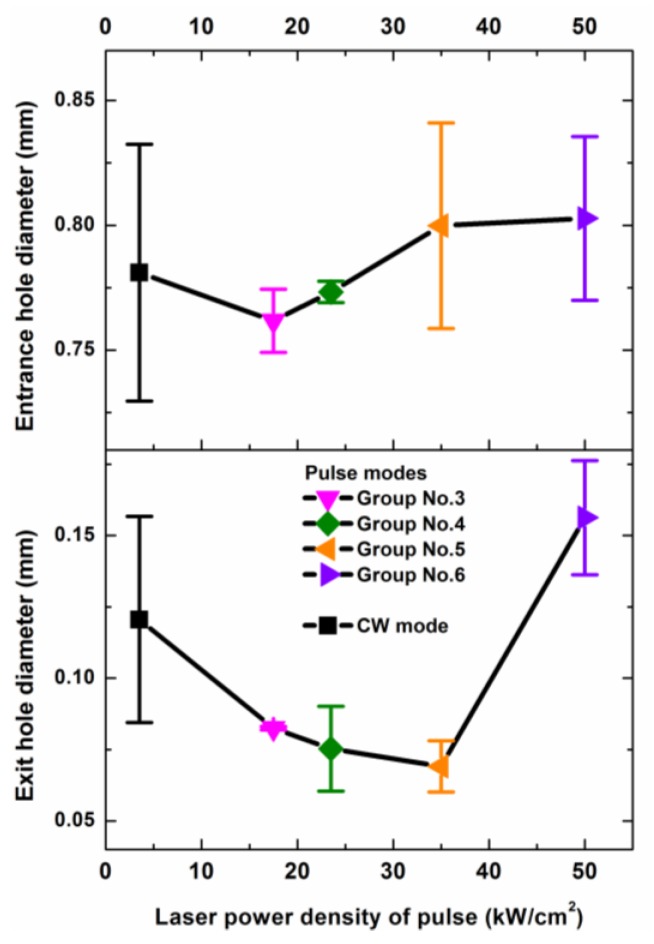
Entrance diameter and exit diameter dependences on the different laser drilling parameters. The laser parameters and the laser irradiation times for the different laser modes are shown in Table 2 and Table 3, respectively.

The entrance hole diameters and the exit hole diameters of the drilled holes with different laser modes are shown in Figure 14. From Figure 14, it can be seen that the difference of the entrance hole diameters for the drilled holes was less than 0.125 mm. In addition, the difference caused by different laser modes was not bigger than the maximum value of the difference caused by the same laser mode. The maximum difference of the exit hole diameters was also less than 0.125 mm. 

Since the holes were considered conical, the tapers of the holes could be calculated with the following expression:

(5) $$\theta_{Taper}=tan^{-1}\left(\;\frac{D_{entra}-D_{exit}}{2h}\;\right),\;h=1\;\mathrm{mm}.$$

**Figure 15:**
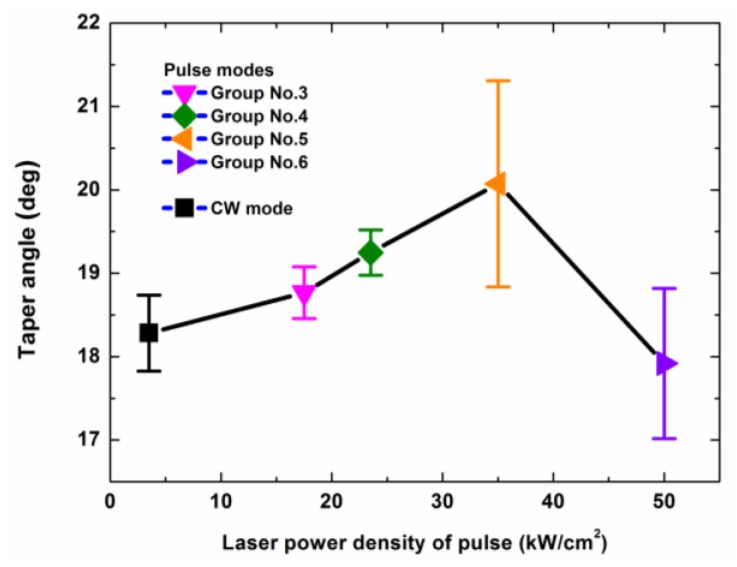
Taper angle dependence on the different laser drilling parameters. The laser parameters and the laser irradiation times for the different laser modes are shown in Table 2 and Table 3, respectively.

The tapers of the drilled holes that were made using different laser modes are shown in Figure 15. From Figure 15, it can be seen that the maximum difference of the tapers was less than 4.5°. Therefore, the drilled holes could be considered similar.

**Figure 16:**
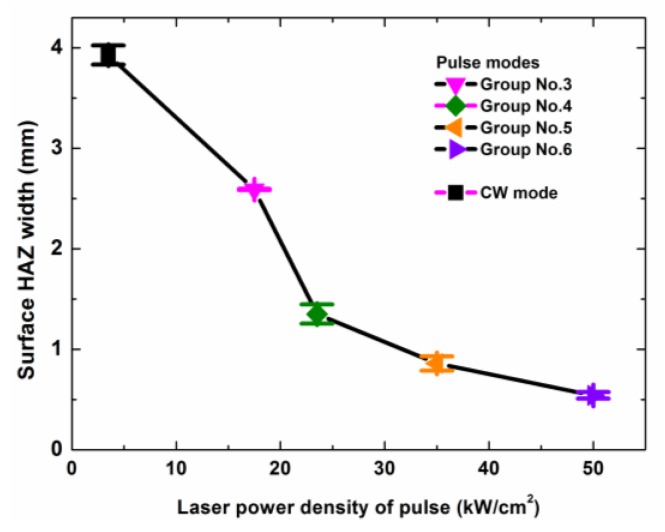
Surface heat affected zone (HAZ) width dependence on the different laser drilling parameters. The laser parameters and the laser irradiation times for the different laser modes are shown in Table 2 and Table 3, respectively.

However, Figure 16 shows that the surface HAZ width increased quickly with the decrease of the peak power density. This occurred because the laser irradiation time that was needed to drill the same hole increased greatly with the decrease of the peak power density, as shown in Table 3. A lower peak power density and a longer irradiation time obviously led to a larger surface HAZ, and the maximum surface HAZ width (3.93 mm) was about seven times the minimum surface HAZ width (0.54 mm), as shown in Figure 16. This fully showed that for the laser output of the same average power density, the ms pulse mode, which had a high peak power density and a low duty cycle, was useful for improving the drilling quality and efficiency by reducing the surface HAZ width, the drilling time, and the laser energy consumption.

## 6. Conclusions

A series of experiments were conducted to investigate the long pulse ms laser drilling of the CFRP composite. For the same average power density of 3.5 kW/cm^2^, it could be found that the drilling time of the ms pulse laser was far lower than that of the CW laser when the peak power density of the ms pulse laser was more than five times that of the CW laser. The drilling time decreased with the increase of the peak power density. When the peak power density of the pulse laser was higher than 23.4 kW/cm^2^, the time of the laser penetration of the composite target decreased sharply. By numerical simulation, it was considered that the large internal gas pressure that resulted from polymer pyrolysis in the composite caused a large amount of mechanical erosion for the CFRP material in the composite to improve the drilling efficiency.

The entrance hole diameter increased with the increase of the laser irradiation time in all of the laser modes. When the peak power density of the pulse laser was higher, the entrance diameter of the drilled hole achieved stability more quickly. The maximum entrance diameter for different pulse laser modes increased with the increase of the peak power density. When the laser irradiation time was long enough, the entrance hole diameter of the CW laser mode could exceed those of some of the pulse laser modes.

The surface HAZ increased with the increase of the laser irradiation time and the decrease of the peak power density. For the drilling of the same hole, the maximum surface HAZ width of 3.93 mm was from the CW laser mode, and the minimum surface HAZ width of 0.54 mm was from the ms pulse laser mode, which had the highest peak power density of 49.8 kW/cm^2^.

The minimum material ablation rate appeared in the ms pulse laser mode, which had the highest peak power density of 49.8 kW/cm^2^ and the shortest laser penetrating time. The maximum material ablation rate appeared in the ms pulse laser mode, which had a middle peak power density of 17.4 kW/cm^2^ and a medium laser penetrating time. This showed that a fast drilling speed could reduce the heat loss from the thermal conduction in order to reduce the HAZ in the composite. Additionally, a medium drilling speed could ensure that the thermal energy had enough time to diffuse along the carbon fibers in order to lead to a large HAZ in the composite.

The results from the experiment and the numerical simulation identified the fact that the ms pulse laser mode, which had a high peak power density and a low duty cycle, was useful for reducing the drilling time, HAZ, and laser energy consumption. It could be concluded that increasing the laser pulse energy and decreasing the pulse duration and duty cycle could improve the laser processing efficiency. The influence of the internal gas pressure on the laser drilling will be studied further in the future.

## Figures and Tables

**Figure A1:**
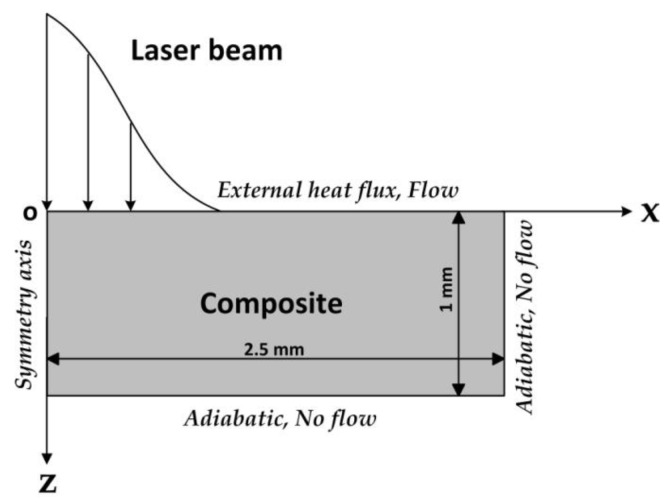
Schematic of the 2D axisymmetric numerical mode describing the laser drilling of the composite.

**Figure A2:**
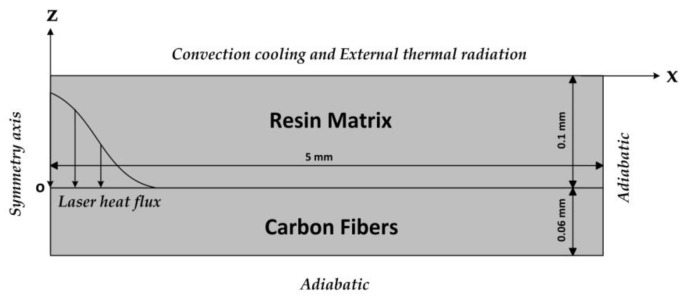
Schematic of the 2D numerical model describing the entrance hole and the surface heat affected zone (HAZ) on the composite surface during the laser drilling.

**Table 1:** Thermo-physical data for a typical carbon fiber reinforced polymer (CFRP) composite.

Physical Data	Matrix	Carbon Fiber Parallel to the Fiber Axis (p)	Carbon Fiber Perpendicular to the Fiber Axis (s)
Density ρ (kg/m^3^)	1250	1850	
Heat conductivity k (W/m/K)	0.2	50	5
Heat capacity $c_{p}$ (J/kg/K)	1200	710	
Sublimation temperature $T_{v}$ (K)	800	3600	
Latent heat $L_{v}$ (kJ/kg)	1000	43,000	

**Table 2:** Laser parameters of the different laser modes performed in the experiment.

Pulse Mode Group No.	Pulse Peak Power (W)	Pulse Peak Power Density (kW/cm^2^)	Pulse Frequency (Hz)	Duty Cycle
1	116	5.8	600	60%
2	180	9.0	400	40%
3	350	17.4	200	20%
4	470	23.4	150	15%
5	700	34.8	100	10%
6	1000	49.8	70	7%
Continuous wave (CW) mode	Mean power (W)	Mean power density (kW/cm^2^)		
	70	3.5		

**Table 3:** Laser irradiation time of drilling the same hole for the different laser modes.

Pulse Mode Group No.	Laser Irradiation Time (s)
3	1.9
4	0.6
5	0.31
6	0.23
Continuous wave (CW) mode	Laser irradiation time (s)
	3.9

## Nomenclature

Glossary of symbols			
A	Pre-exponential factor, s^−1^	Greek symbols	
$c_{p}$	Heat capacity, J/(kg·K)	$\Delta h_{pyr\left(g\right)}$	Heat of pyrolysis reaction, J/kg
$D_{entra}$	Entrance hole diameter, m	δT	Temperature transition zone half width of gasification process, K
$D_{exit}$	Exit hole diameter, m	ε	Emissivity of carbon-phenolic
$d_{f}$	Diameter of carbon fiber, m	$\theta_{Taper}$	Taper of drilled hole
dT	Temperature transition zone half width of latent heat of gasification, K	K	Permeability, m^2^
$E_{a}$	Activation energy, J/mol	$\mu_{g}$	Gas kinematic viscosity, Pa·s
H	Transition function	v	Gas velocity, m/s
h	Thickness of composite, m	ρ	Density, Kg/m^3^
$h_{conv}$	Surface convection coefficient, $W/(m^{2}\cdot K)$	σ	Stefan-Boltzman constant, W/(m^2^·K^4^)
$I_{0}$	Laser peak power density, W/cm^2^	τ	Duty cycle
$I_{s}$	Laser heat fluence with Gauss distribution in space, W/cm^2^		
k	Thermal conductivity, W/(m·K)	Superscripts	
$k_{p}$	Thermal conductivity of composite parallel to fiber axis, W/(m·K)	*air*	Air
$k_{s}$	Thermal conductivity of composite perpendicular to fiber axis, W/(m·K)	*char*	Carbon residue
$L_{v}$	Latent heat of mass gasification, J/kg	*fiber*	Carbon fiber
${ℳ}_{g}$	Molecular weight of pyrolysis gas, g/mol	*gas*	Pyrolysis gas
m	Mass, kg	*poly*	Polymer resin
N	Number of laser pulses		
*n*	Zero or positive integer		
$n_{a}$	Reaction order		
P	Gas pressure, Pa		
$P_{0}$	Initial internal gas pressure, Pa		
$P_{ref}$	Ambient pressure, Pa	Subscripts	
R	Ideal gas constant, J/(mol·K)		
$r_{0}$	Spot radius, m	*e*	End state after reaction
T	Temperature, K	*eff*	Effective parameter
$T_{0}$	Initial temperature, K	o	Original state before reaction
$T_{ref}$	Ambient temperature, K	*sim*	Simulation value
$T_{v}$	Gasification temperature of carbon, K		
$T_{w}$	Temperature of the upper surface, K		
$t_{Laser}$	Laser penetration time, s		
$t_{pulse}$	Pulse duration, s		
V	Volume fraction		
$W_{HAZ}$	Surface HAZ width, m		
*x, y, z*	Cartesian coordinate system		

## Appendix A

### Appendix A.1. Estimation of the Laser Penetration Time

In Section 3.1, the laser penetration time could be calculated as follows:

(3) $$t_{Laser}=\frac{\rho (L_{v}+C_{p}\left(T_{v}-T_{0}\right))h}{2I_{0}\tau }.$$

In Reference [20], the effective total interaction time, $t_{HeatLoad}$, could be calculated as follows:

(A1) $$t_{HeatLoad}=\frac{E_{heat}+E_{Lv}}{P_{Laser}}=\frac{\ell_{Proc}\cdot \rho \cdot \left(c_{p}\cdot \left(T_{sub}-T_{0}\right)+L_{v}\right)}{P_{Laser}∕A_{Spot}},$$

where $P_{Laser}$, $\ell_{Proc}$, $A_{Spot}$, and $T_{sub}$ were the laser power, the process-depth, the spot area, and the sublimation temperature, respectively.

The two equations are based on the energy balance in the process of the carbon fiber sublimation in which the heat diffuse along the fibers is neglected. In Equation (3), the thickness of the specimen h represents the $\ell_{Proc}$ of Equation (A1) and the $T_{v}$ represents the $T_{sub}$ of Equation (A1). In the reference, the laser intensity (power density) $I_{Laser}=P_{Laser}∕A_{Spot}$. In this study, the Gaussian laser intensity could be written as:

(A2) $$I_{s}=2I_{0}e^{-\frac{2r^{2}}{r_{0}^{2}}}f_{i}\left(t\right),$$

where r, $r_{0}$, $I_{0}$ were the distance from the spot center, the spot radius, and the average laser intensity in the spot. The pulse peak power density of the ms laser and the mean power density of the CW laser as shown in Table 2 were calculated with the expression: $I_{0}=P_{Laser}∕A_{Spot}$.

From Equation (A2), it could be found that the maximum value of the laser intensity ($2I_{0}$) was in the center of the laser spot ($r_{0}=0$). In CW laser mode, the duty cycle τ = 1 and $f_{i}\left(t\right)=1$. Therefore, the laser intensity in the center of the laser spot kept $2I_{0}$. In the pulse laser mode, $f_{i}\left(t\right)$ was a periodic function of square wave, as shown below:

(A3) $$f_{i}\left(t\right)=1,\;n\frac{t_{pulse}}{\tau }\leq t\leq n\frac{t_{pulse}}{\tau }+t_{pulse},$$

where n was zero or a positive integer and the pulse duration $t_{pulse}=1\;\mathrm{ms}$. Additionally, the duty cycle τ was as shown in Table 2. Therefore, the average laser intensity in the center of the laser spot could be calculated as $2I_{0}\tau$. So $2I_{0}\tau$ could represent the $I_{Laser}$ of Equation (A1). Therefore, Equation (3) could be used to estimate the laser penetration time in Section 3.1.

### Appendix A.2. Model Formulation for the Internal Gas Pressure

For Section 4.1, in order to predict the temperature field and the internal gas pressure in the composite during the laser drilling, a two-dimensional axisymmetric finite element model was established. To simplify the calculation, the composite could be considered a homogeneous anisotropic block [24,25]. The schematic of the model is shown in Figure A1.

Additionally, some assumptions were adopted, as follows:

(1) The laser energy was completely absorbed on the surface of the composite.
(2) The possible oxidation reaction and the material erosion on the composite surface were neglected.
(3) The polymer matrix degraded into the carbon residue and pyrolysis gas directly via heating.
(4) The pyrolysis gas was chemically non-reactive and the ideal gas equation of state could be used.
(5) The pyrolysis gas was always in thermal equilibrium with the nearby solid.
(6) The volume change and the thermal expansion of the material were neglected.

Therefore, during the laser drilling, the polymer matrix that experienced pyrolysis contained three types of components: polymer resin, decomposition gases, and carbon residue (char). The $\rho c_{p}$ of the composite could be written as [26]:

(A4) $$\rho c_{p}=V^{poly}\rho^{poly}c_{p}{}^{poly}+V^{char}\rho^{char}c_{p}{}^{char}+V^{fiber}\rho^{fiber}c_{p}{}^{fiber}+V^{gas}\rho^{gas}c_{p}{}^{gas},$$

where $V^{i}$ represented the volume fraction of component *i* in a unit volume. $V^{i}$ was given with reference to the mass of component *i* ($m^{i}$) and the constant density of component *i* ($\rho^{i}$) by considering

(A5) $$V^{i}=\frac{m^{i}}{\rho^{i}}.$$

In addition, $V^{gas}\rho^{gas}c_{p}{}^{gas}$ could be neglected because $\rho^{gas}$ was much smaller than $\rho^{poly}$, $\rho^{char}$, and $\rho^{fiber}.$ The quantity $R_{a}$ is the ratio of the completely pyrolyzed matrix to the virgin matrix as follows:

(A6) $$R_{a}=\frac{m_{e}^{char}}{m_{o}^{poly}},$$

where the subscripts ‘o’ and ‘e’ denote the original state and the end state, respectively. $R_{a}=0.17$ was used [27]. Hence, the mass transforming relationship of the polymer resin and char could be given by:

(A7) $$\frac{dm^{char}}{dt}=R_{a}\frac{dm^{poly}}{dt}.$$

The decomposition gases volume fraction $V^{gas}$ could be calculated with the following expression:

(A8) $$V^{gas}=1-V^{poly}-V^{char}-V^{fiber}.$$

The initial volume fraction of gas $V_{o}^{gas}$ was assumed to be 0.003 in this model.

The governing equation of the thermal transport through the composite could be written as follows [24,25,28]:

(A9) $$\rho c_{p}\frac{dT}{dt}=\nabla \cdot \left(k\nabla T\right)+\Delta \;h_{pyr\left(g\right)}\frac{dm^{poly}}{dt}-\nabla \;\left(v^{gas}\rho^{gas}c_{p}{}^{gas}T\;\right),$$

where $\Delta \;h_{pyr\left(g\right)}$ and $v^{gas}$ were the heat of decomposition and the gas velocity, respectively. The second term on the right-hand side of Equation (A9) represents the energy consumption due to polymer pyrolysis. The third term on the right-hand side represents a contribution of the gas convection to the internal energy. The thermal conductivity perpendicular to the incident laser beam could be considered with the following expression [18,24]:

(A10) $$k_{x}=k_{y}=V^{poly}k^{poly}+V^{char}k^{char}+V^{fiber}k_{p}^{fiber}+V^{gas}k^{gas}.$$

The thermal conductivity in the thickness direction $k_{z}$ is shown in Table A1 [29]. Additionally, some material thermal parameters used in the model are shown in Table 1, and the others are shown in Table A2 [18,24].

**Table A1:** Carbon-phenolic thermal conductivity perpendicular to the fiber axis.

T (K)	$k_{z}$ (W/(cm·K))
300	0.0056
422	0.0076
533	0.0085
811	0.0054
1367	0.0077
1922	0.0099
3500	0.0222

**Table A2:** Some thermal parameters of the components in the composite.

Parameter	Symbol	Value	Units
Thermal conductivity of the polymer resin [18]	$k^{poly}$	0.2	W/(m·K)
Density of the polymer resin [24]	$\rho^{poly}$	900	kg/m^3^
Heat capacity of the polymer resin [24]	$c_{p}{}^{poly}$	2500	J/(kg·K)
Thermal conductivity of the carbon residue [24]	$k^{char}$	0.2	W/(m·K)
Density of the carbon residue [24]	$\rho^{char}$	1300	kg/m^3^
Heat capacity of the carbon residue [24]	$c_{p}{}^{char}$	1589	J/(kg·K)
Thermal conductivity of the pyrolysis gas [24]	$k^{gas}$	0.025	W/(m·K)
Density of the pyrolysis gas [24]	$\rho^{gas}$	1.997	kg/m^3^
Heat capacity of the pyrolysis gas [24]	$c_{p}{}^{gas}$	720	J/(kg·K)
The heat of decomposition [24]	$\Delta h_{pyr\left(g\right)}$	9 × 10^5^	J/kg
Thermal conductivity of the carbon fiber parallel to the fiber axis [18]	$k_{p}^{fiber}$	50	W/(m·K)
Thermal conductivity of the carbon fiber perpendicular to the fiber axis [18]	$k_{s}^{fiber}$	5	W/(m·K)
Density of the carbon fiber [18]	$\rho^{fiber}$	1850	kg/m^3^
Heat capacity of the carbon fiber [18]	$c_{p}{}^{fiber}$	710	J/(kg·K)

In Table A2, the parameters of CO_2_ were used as the thermal parameters of the pyrolysis gases.

The rate of polymer resin pyrolysis could be roughly described using an Arrhenius equation with the form of [24,25]:

(A11) $$\frac{dm^{poly}}{dt}=-Am_{o}^{poly}{\left(\frac{m^{poly}+m^{char}-m_{e}^{char}}{m_{o}^{poly}}\right)}^{n_{a}}exp\left(-\frac{E_{a}}{RT}\;\right),$$

where A, $n_{a}$, $E_{a}$, R were the pre-exponential factor, the reaction order, the activation energy, and the ideal gas constant, respectively. In the model, A is 3.15 × 10^11^ s^−1^, $n_{a}$ is 1.344, $E_{a}$ is 1.8173 × 10^5^ J/mol, and R is 8.31 J/(mol·K) [24].

The relationship of the mass conservation of the internal gas was given by the continuity equation as follows:

(A12) $$-\frac{dm^{poly}}{dt}=\nabla \cdot \left(\rho^{gas}v^{gas}\right)+\frac{dm^{gas}}{dt}.$$

The left-hand side of Equation (A12) represents the mass of the gases produced by matrix decomposition. The first term on the right-hand of Equation (A12) represents the gas mass convection through the porous material. The second term on the right-hand is the time rate of the change of gas storage in the composite. This term is commonly negligible. And $v^{gas}$ was given by the Darcy filtration law as follows [24,25,30]:

(A13) $$v^{gas}=-\frac{K}{\mu_{g}}\nabla \;P,$$

where K, $\mu_{g}$, and P were the permeability, the gas kinematic viscosity, and the gas pressure inside the decomposing composite, respectively. Additionally, the calculation equation of the permeability could be written as follows:

(A14) $$K=\frac{d_{f}{}^{2}V^{gas}{}^{3}}{C{\left(1-V^{gas}\right)}^{2}},$$

where $d_{f}$ was the diameter of a single carbon fiber, defined as 6 μm and *C* = 144 [27,31]. The kinematic viscosity that was used in the model was assumed to be 3 × 10^−5^ Pa·s. Because the trapped gas was assumed to be an ideal gas, the gas pressure could be calculated with the following expression:

(A15) $$P=\frac{m^{gas}RT}{{ℳ}_{g}V^{gas}},$$

where ${ℳ}_{g}$ was used as the average molecular weight of CO_2_ 44 g/mol.

In addition, when the temperature of the composite material exceeded the vaporization point of carbon ($T_{v}$ = 3600 K), a simplified sublimation mechanism was used, as shown below:

(A16) $$H=\{\begin{array}{c} 1\\ \frac{T-\left(T_{v}+dT\right)}{\delta T}\\ 0 \end{array},\;\begin{array}{c} T>\left(T_{v}+dT\right)+\delta T\\ \left(T_{v}+dT\right)-\delta T<T<\left(T_{v}+dT\right)+\delta T\\ T<\left(T_{v}+dT\right)-\delta T \end{array},$$

(A17) $$\rho_{eff}=\left(-\rho +\rho^{air}\right)H+\rho ,$$

(A18) $$k_{p}{}_{eff}=\left(-k_{p}+k^{air}\right)H+k_{p},$$

(A19) $$c_{p}{}_{eff}=\left[c_{p}{}^{air}-\left(c_{p}+L_{v}D_{v}\right)\right]H+c_{p}+L_{v}D_{v},$$

(A20) $$D_{v}=\frac{exp\left[{\left(T-T_{v}\right)}^{2}/{\left(dT\right)}^{2}\right]}{\sqrt{\pi }dT},$$

where the latent heat of carbon sublimation $L_{v}$ was added to the equivalent heat capacity $c_{p}{}_{eff}$ of the composite material [32,33]. Here, dT=50 K and δT=50 K. To simulate the process of laser irradiation on the new composite surface after the original surface ablated, a large thermal conductivity in the thickness direction was used, as shown below:

(A21) $$k_{s}{}_{eff}=k_{L}H+k_{s}.$$

In this model, $k_{L}=$ 5 × 10^3^ W/(m·K). The $k_{p}$, $k_{s}$, ρ, and $c_{p}$ terms could be replaced with $k_{p}{}_{eff}$, $k_{s}{}_{eff}$, $\rho_{eff}$, and $c_{p}{}_{eff}$, respectively. Then, the numerical model could describe the two processes of the thermal decomposition in the composite and the sublimation on the surface at the same time.

Because the pyrolysis gases did not absorb and shield the laser energy, the laser energy received by the composite surface was equal to the output energy of the laser. Although the pyrolysis gases that were released from the composite surface changed the ambient temperature and the convective heat transfer on the surface [29,34,35,36], the effect of the pyrolysis gases on the surface heat exchange was much less than the effect of the absorbed laser fluence in the model was. Therefore, the boundary conditions of the laser drilling process could be represented as follows:

(A22) $$-k_{s,eff}\frac{\partial T}{\partial z}=I_{s}-h_{conv}\left(T_{w}-T_{ref}\right)-\varepsilon \sigma \left(T_{w}^{4}-T_{ref}^{4}\right),\;z=0,$$

(A23) $$I_{s}=2I_{0}e^{-\frac{2\left(x^{2}+y^{2}\right)}{r_{0}^{2}}}f_{i}\left(t\right),$$

(A24) $$\frac{\partial T}{\partial z}=0,z=1\;\mathrm{mm};\;\frac{\partial T}{\partial x}=0,x=2.5\;\mathrm{mm},$$

where the spot radius $r_{0}=0.8\;\mathrm{mm}$, the heat transfer coefficient $h_{conv}=50\;W/m^{2}\cdot K$, the emissivity of carbon-phenolic ε=0.85, and the Stefan-Boltzmann constant $\sigma =5.67\times 10^{-8}\;W/m^{2}\cdot K^{4}$. $T_{ref}$ is the ambient temperature and $T_{w}$ is the temperature of the irradiated surface. The first term on the right-hand side of Equation (A22) represents the laser heat fluence with a Gauss distribution in space. The second term on the right-hand side of Equation (A22) represents the air convection cooling on the surface, and the third term on the right-hand side of Equation (A22) represents the heat exchange between the heated surface and surroundings.

The above mathematical model was achieved numerically through the finite element method (FEM) with the help of the business software COMSOL 4.2. In COMSOL 4.2, Equations (A9),(A11),(A12) could be calculated using the Heat Transfer Module, the Chemical Reaction Engineering Module, and the Computational Fluid Dynamics (CFD) Module, respectively. These three main governing equations were coupled by the variables of $m^{i}$, T, and $v^{gas}$. The geometry of 2.5 mm × 1 mm, as shown in Figure A1, was divided by a non-uniform mapped mesh. The external heat flux defined by Equation (A22) was positioned at the top surface (*z* = 0), which generated a large temperature gradient near the laser center. Hence, the distribution of the elements in the mesh was dense at the laser radiation area and the distribution gradually became sparse from the Point o (0,0) to the outside in a fixed ratio. In this model, the minimum spatial element size was less than 9 × 10^−7^ m. The edge boundaries of *x =* 2.5 mm and *z =* 1 mm were adiabatic (Equation (A24)). The pyrolysis gases could only flow out from the edge boundary of *z =* 0. The initial temperature $T_{0}$ was assumed to be 300 K, and the initial internal gas pressure $P_{0}$ was assumed to be 1 atm. The constant ambient temperature $T_{ref}$ was assumed to be 300 K, and the constant ambient pressure $P_{ref}$ was assumed to be 1 atm. The initial volume fractions of the composite components were $V_{o}^{poly}=0.3$, $V_{o}^{fiber}=0.697$, and $V_{o}^{gas}=0.003$.

### Appendix A.3. Model Formulation for the Entrance Hole Diameter and the Surface HAZ Width

For Section 4.2, to accurately predict the entrance hole diameter and the surface HAZ, a new geometric model was proposed, as shown in Figure A2. In this model, the resin matrix layer with a 0.1 mm thickness and the carbon fibers layer with a 0.06 mm thickness were separated. The Point o (0,0) was at the interface of the two layers, as shown in Figure A2.

In the resin matrix layer, the governing equations were simplified as follows:

(A25) $$\rho_{eff}^{poly}c_{p}{}_{eff}^{poly}\frac{dT}{dt}=\nabla \cdot \left(k_{eff}^{poly}\nabla T\right),$$

(A26) $$\rho_{eff}^{poly}=\left(-\rho^{poly}+\rho^{air}\right)H^{poly}+\rho^{poly},$$

(A27) $$k_{eff}^{poly}=\left(-k^{poly}+k^{air}\right)H^{poly}+k^{poly},$$

(A28) $$c_{p}{}_{eff}^{poly}=\left[c_{p}{}^{air}-\left(c_{p}{}^{poly}+L_{v}^{poly}D_{v}^{poly}\right)\right]H^{poly}+c_{p}{}^{poly}+L_{v}^{poly}D_{v}^{poly},$$

(A29) $$H^{poly}=\{\begin{array}{c} 1\\ \frac{T-850\;K}{50\;K}\\ 0 \end{array}\;\;\begin{array}{c} T>850\;K+50\;K\\ 850\;K-50\;K<T<850\;K+50\;K\\ T<850\;K-50\;K \end{array},$$

(A30) $$D_{v}^{poly}=\frac{exp\left[{\left(T-800\;K\right)}^{2}/{\left(50\;K\right)}^{2}\right]}{\sqrt{\pi }\;50\;K}.$$

In the carbon fibers layer, the governing equations were simplified as follows:

(A31) $$\rho_{eff}^{fiber}c_{p}{}_{eff}^{fiber}\frac{dT}{dt}=\nabla \cdot \left(k_{eff}^{fiber}\nabla T\right),$$

(A32) $$\rho_{eff}^{fiber}=\left(-\rho^{fiber}+\rho^{air}\right)H^{fiber}+\rho^{fiber},$$

(A33) $$c_{p}{}_{eff}^{fiber}=\left[c_{p}{}^{air}-\left(c_{p}{}^{fiber}+L_{v}^{fiber}D_{v}^{fiber}\right)\right]H^{fiber}+c_{p}{}^{fiber}+L_{v}^{fiber}D_{v}^{fiber},$$

(A34) $$k_{p,eff}^{fiber}=\left(-k_{p}^{fiber}+k^{air}\right)H^{fiber}+k_{p}^{fiber},$$

(A35) $$k_{s,eff}^{fiber}=H^{fiber}\;5\times 10^{3}\;W/\left(m\;K\right)+k_{s}^{fiber},$$

(A36) $$H^{fiber}=\{\begin{array}{c} 1\\ \frac{T-3650\;K}{50\;K}\\ 0 \end{array}\;\;\begin{array}{c} T>3650\;K+50\;K\\ 3650\;K-50\;K<T<3650\;K+50\;K\\ T<3650\;K-50\;K \end{array},$$

(A37) $$D_{v}^{fiber}=\frac{exp\left[{\left(T-3600\;K\right)}^{2}/{\left(50\;K\right)}^{2}\right]}{\sqrt{\pi }\;50\;K}.$$

This model only considered the entrance hole diameter and the surface HAZ width, so the pyrolysis process of the polymer resin could be neglected. Therefore, the numerical model only needed to use the Heat Transfer Module of COMSOL. Additionally, because the epoxy resin was transparent for the NIR laser [8,38] and the absorption depth of the 1064 nm laser inside of carbon fiber varied in the range of 74 to 270 nm [39,40], the laser energy that was absorbed at the upper surface of the carbon fibers layer was considered in this model. Thus, the boundary conditions could be written as follows:

(A38) $$k_{eff}^{poly}\frac{\partial T}{\partial z}=h_{conv}\left(T_{w}-T_{ref}\right)+\varepsilon \sigma \left(T_{w}^{4}-T_{ref}^{4}\right),z=0.1\;\mathrm{mm},\;$$

(A39) $$-k_{s,eff}^{fiber}\frac{\partial T}{\partial z}=I_{s},z=0,$$

(A40) $$\frac{\partial T}{\partial z}=0,\;z=-0.06\;\mathrm{mm}\;\frac{\partial T}{\partial x}=0,x=5\;\mathrm{mm}.$$

In addition, the distribution of elements in the mesh was dense at the boundary of *z =* 0, and the distribution gradually became sparse from the laser center of the interface to the outside in a fixed ratio. In this model, the minimum spatial element size was less than 2.5 × 10^−7^ m. The initial temperature $T_{0}$ and the constant ambient temperature $T_{ref}$ were assumed to be 300 K. In the resin matrix layer, $V_{o}^{poly}=1$. In the carbon fibers layer, $V_{o}^{fiber}=1$. And the other calculation parameters were shown in Table 1, Table A1 and Table A2.

In all of the calculation time, the entrance hole diameter from the numerical simulation was defined as follows:

(A41) $$D_{entra,\;sim}=2\;max\left(\sqrt{x^{2}+y^{2}}\right)\times \left(T\geq 3650\;K\right),z=0.$$

And the surface HAZ width from the numerical simulation was defined as follows:

(A42) $$W_{HAZ,\;sim}=max\left(\sqrt{x^{2}+y^{2}}\right)\times \left(T\geq 850\;K\right)-D_{entra,\;sim}/2,z=0.1\;\mathrm{mm}.$$
